# Myocardial Infarction After High-Dose Catecholamine Application—A Case Report From an Experimental Imaging Study

**DOI:** 10.3389/fcvm.2020.580296

**Published:** 2020-11-19

**Authors:** Niklas Beyhoff, David Lohr, Arne Thiele, Anna Foryst-Ludwig, Robert Klopfleisch, Laura M. Schreiber, Ulrich Kintscher

**Affiliations:** ^1^Charité - Universitätsmedizin Berlin, Corporate Member of Freie Universität Berlin, Humboldt-Universität zu Berlin, and Berlin Institute of Health, Institute of Pharmacology, Center for Cardiovascular Research, Berlin, Germany; ^2^DZHK (German Centre for Cardiovascular Research), Partner Site Berlin, Berlin, Germany; ^3^Berlin Institute of Health (BIH), Berlin, Germany; ^4^Chair of Molecular and Cellular Imaging, Comprehensive Heart Failure Center, University Hospital Würzburg, Würzburg, Germany; ^5^Department of Veterinary Pathology, College of Veterinary Medicine, Freie Universität Berlin, Berlin, Germany

**Keywords:** myocardial infarction, catecholamines, speckle tracking, diffusion tensor imaging, magnetic resonance imaging, case report, heart failure, echocardiography

## Abstract

Although heart failure following myocardial infarction (MI) represents a major health burden, underlying microstructural and functional changes remain incompletely understood. Here, we report on a case of unexpected MI after treatment with the catecholamine isoproterenol in an experimental imaging study in mice using different state-of-the-art imaging modalities. The decline in cardiac function was documented by ultrahigh-frequency echocardiography and speckle-tracking analyses. Myocardial microstructure was studied *ex vivo* at a spatial resolution of 100 × 100 × 100 μm^3^ using diffusion tensor magnetic resonance imaging (DT-MRI) and histopathologic analyses. Two weeks after ISO treatment, the animal showed an apical aneurysm accompanied by reduced radial strain in corresponding segments and impaired global systolic function. DT-MRI revealed a loss of contractile fiber tracts together with a disarray of remaining fibers as corresponding microstructural correlates. This preclinical case report provides valuable insights into pathophysiology and morphologic–functional relations of heart failure following MI using emerging imaging technologies.

## Introduction

Although heart failure following myocardial infarction (MI) represents a major health burden, underlying structural/functional relationships remain incompletely understood (1, 2). While adverse cardiac remodeling after MI is considered to directly affect the mechanical and electrical properties of the heart (3–5), the exact impact of microstructural changes on myocardial function remains unclear. Here, we report on a case of unexpected MI after treatment with the catecholamine isoproterenol (ISO) in an experimental imaging study in mice correlating a comprehensive set of functional parameters with detailed histopathology and diffusion tensor magnetic resonance imaging (DT-MRI).

## Case Description

**Figure 1:**
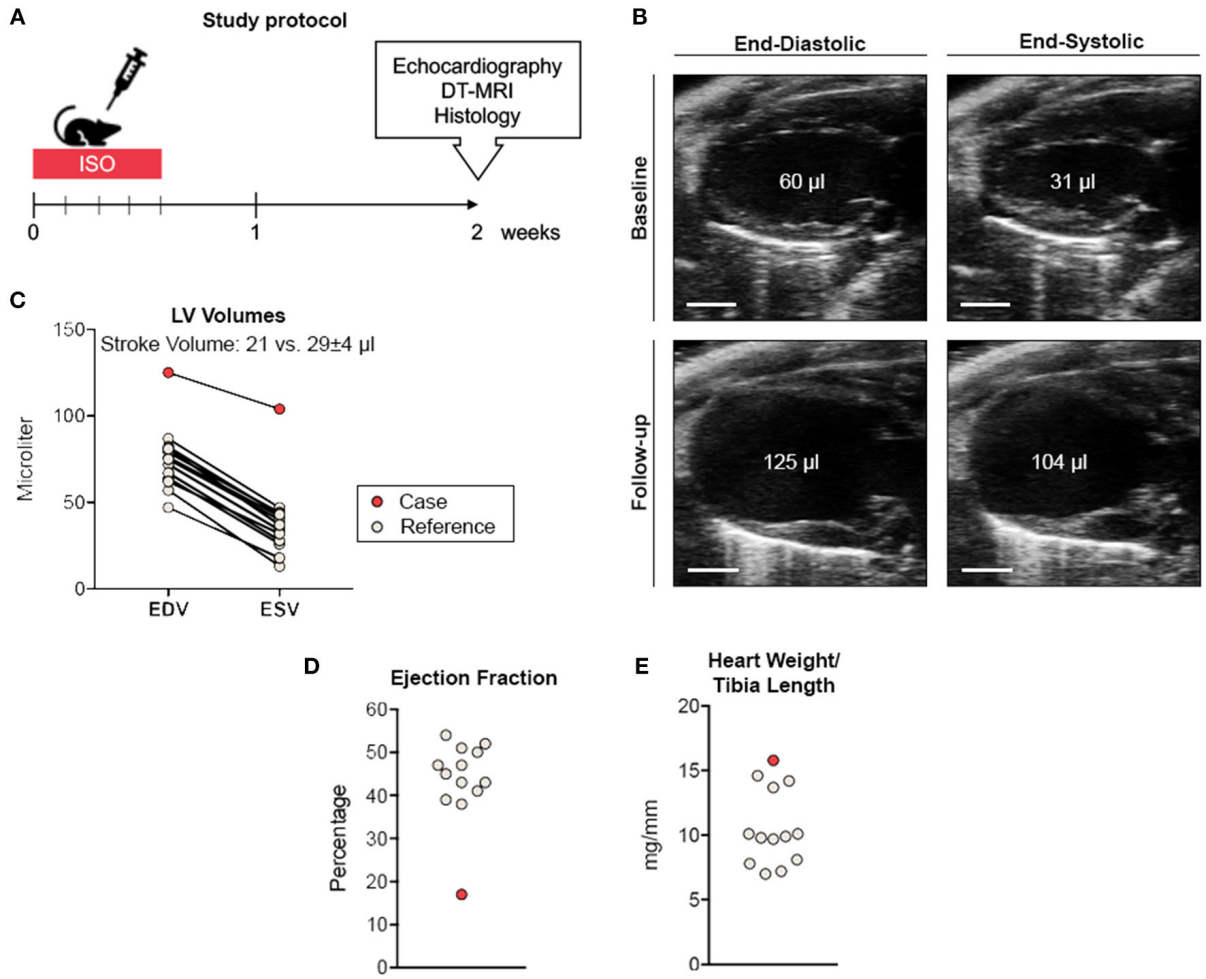
Study protocol and cardiac phenotyping. **(A)** Study protocol. **(B)** Parasternal long-axis view during end-diastole and end-systole before (upper panel) and 2 weeks after ISO treatment (lower panel). Scale bar represents 2 mm. **(C)** Comparative volumetry indicating increased left ventricular volumes accompanied by reduced stroke volume. **(D)** Ejection fraction analysis. **(E)** Normalized heart weights indicating cardiac enlargement. Remaining ISO-treated animals (*n* = 12) served as reference. EDV, end-diastolic volume; ESV, end-systolic volume.

**Figure 2:**
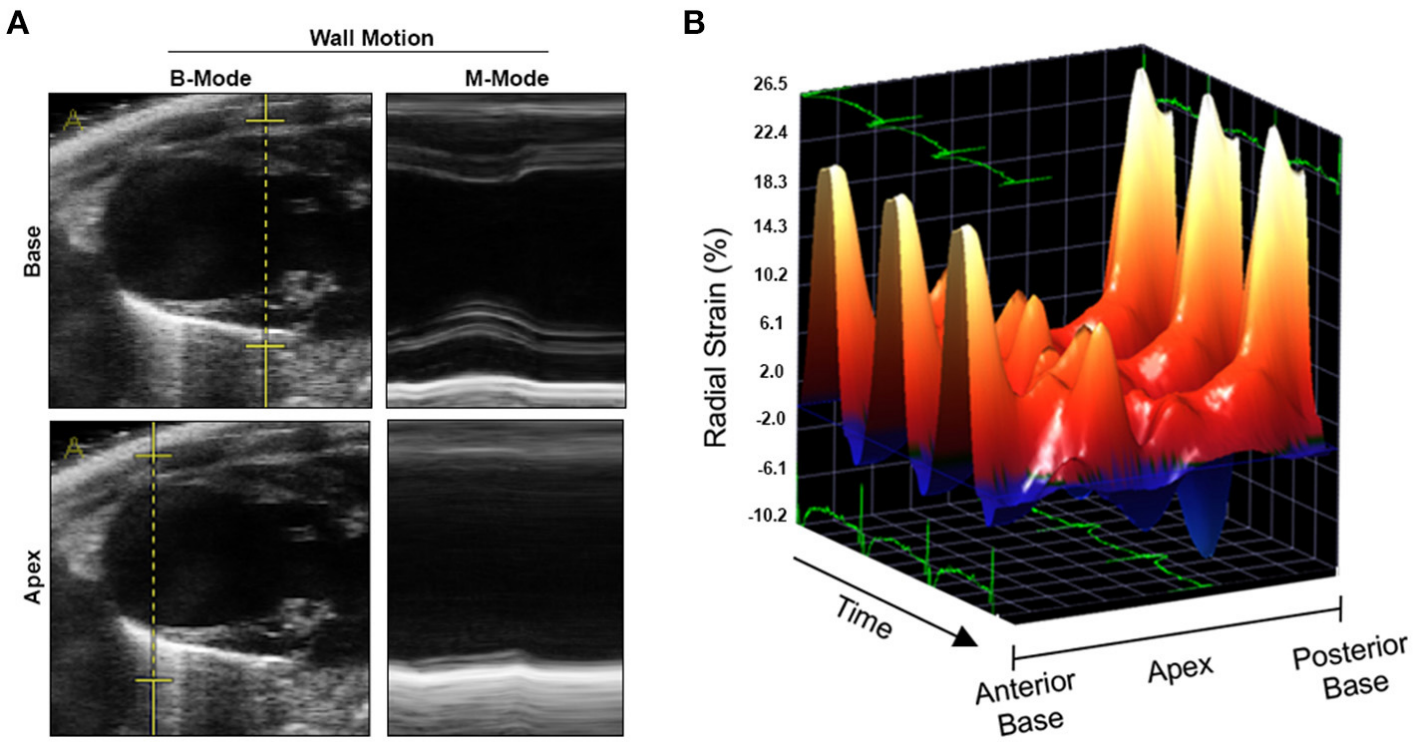
Wall motion analyses by conventional and speckle-tracking echocardiography. **(A)** Reconstructed M-modes at basal (upper panel) and apical level (lower panel) indicating apical akinesia. **(B)** Three-dimensional reconstruction of radial strain during three cardiac cycles. Three-dimensional Cartesian coordinate system mapping radial strain, time/cardiac cycles, and myocardial segments derived from the cardiac long axis (from anterior base over apex to posterior base). Radial strain in midmyocardial and apical segments (both anterior and posterior) was markedly lower as compared to basal segments.

We conducted an experimental imaging study to characterize myocardial microstructure and function in a murine model of circumscribed subendocardial damage whose results have been published previously (6). Briefly, male 129/Sv mice (6–8 weeks old) received subcutaneous injections of 25 mg/kg ISO or saline as placebo control for four consecutive days according to a standard protocol (Figure 1A) (6). The case animal was randomized to the ISO group. During baseline echocardiography prior to treatment, all animals showed comparable parameters of cardiac function and ventricular dimensions. At baseline, the case animal had a left ventricular ejection fraction of 48% without any evidence of regional wall motion abnormalities (Figure 1B and Supplementary Video 1). First differences became apparent after the first injection, where it required a longer recovery period than its littermates. Recovery time was also slightly prolonged after the second to fourth injection. Typically, ISO does not cause chronic alterations of ventricular dimensions and systolic function in this model (6). However, echocardiography 2 weeks after final injection revealed wall thinning and a pronounced aneurysm of the apex of the left ventricle (LV) resulting in massively increased LV volumes and markedly reduced global systolic function (Figures 1B–D). Cardiac enlargement was also evident from indexed heart weight obtained during necropsy (Figure 1E). Apical segments were akinetic, whereas the base appeared to contract normally (Figure 2A and Supplementary Video 2). During speckle-tracking echocardiography, the same segmental differences were observed in reduced apical but preserved basal radial strain of the LV (Figure 2B). The ISO-mediated decline in longitudinal deformation indices (7) was substantially more pronounced in this animal (global longitudinal strain: −4.6 vs. −12.5% ± 1.9%; global longitudinal strain rate: −1.8 vs. −3.9 ± 0.7^−s^).

**Figure 3:**
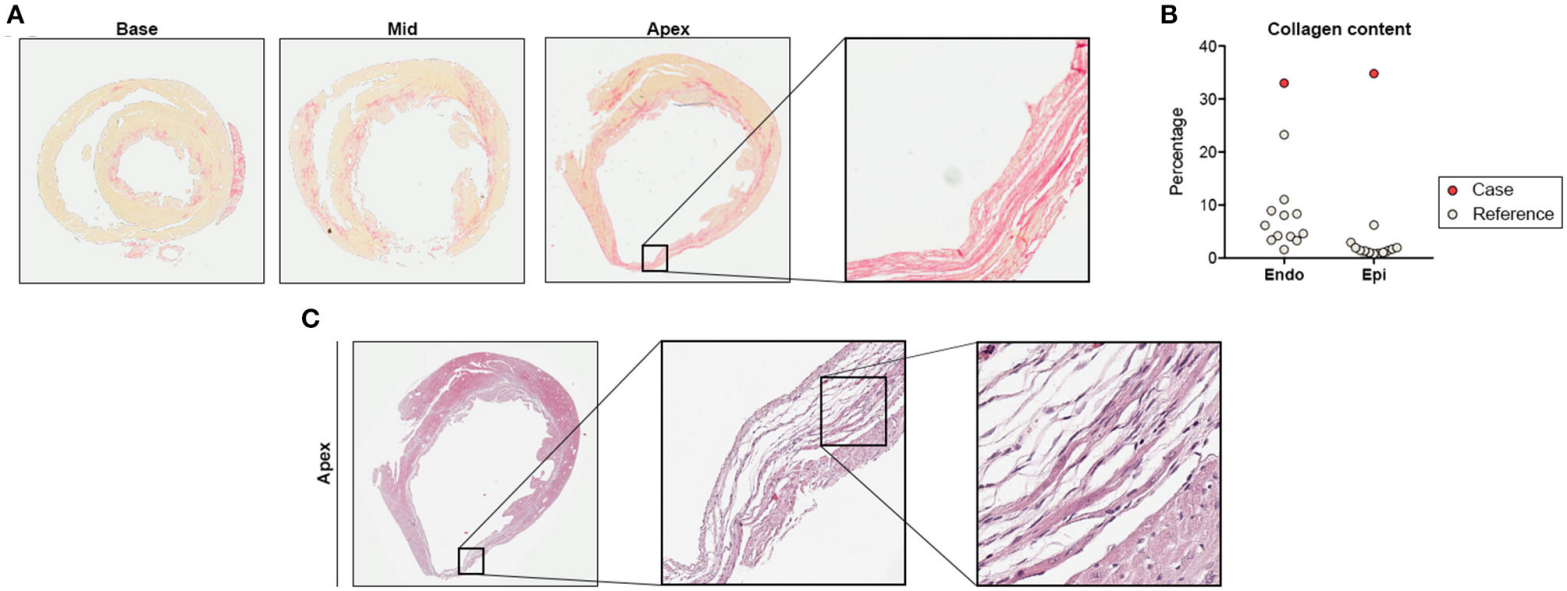
Histological analysis. **(A)** Cardiac cross-sections at basal, midmyocardial, and apical level with exemplary region of interest showing transmural scarring (Picrosirius red staining for detection of collagen fibers). **(B)** Collagen quantification in subendocardium (Endo) and subepicardium (Epi). **(C)** Detailed histology of the apical scar presented in **(A)** indicating replacement fibrosis in response to cardiomyocyte loss (hematoxylin-eosin stain). Remaining ISO-treated animals (*n* = 12) served as reference.

Histological analyses showed subendocardial fibrosis within basal and midmyocardial sections of the LV (Figure 3A). Fibrotic lesions in the apex exceeded the subendocardial layer resulting in transmural scarring and aneurysmatic wall thinning (Figures 3A,B). In contrast, the remaining ISO-treated animals showed circumscribed subendocardial collagen accumulation, as expected in this model (Figure 3B) (6, 7). Histopathology of the apical scar revealed replacement fibrosis in response to a profound loss of cardiomyocytes (Figure 3C).

**Figure 4:**
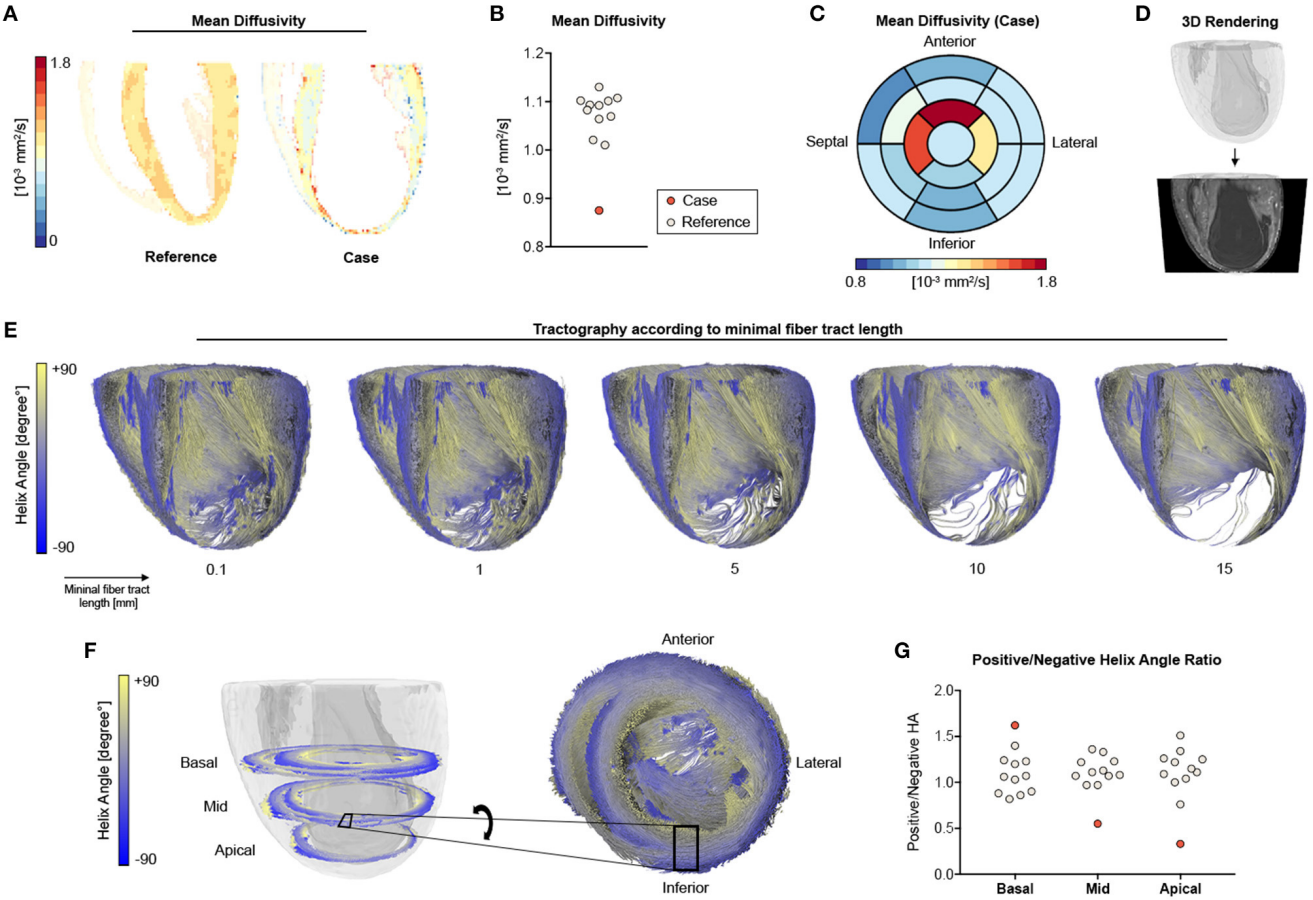
DT-MRI analysis. **(A)** Long-axis mean diffusivity for a representative heart of the ISO group (reference) and the infarcted heart (case). **(B)** Quantitative analyses of mean diffusivity in the left ventricle. **(C)** Bull's-eye plot for mean diffusivity of the infarcted heart. **(D)** Whole-heart volume rendering and respective surface cut for tractography visualization in **(E)** tractography of the main eigenvector using varying minimal fiber bundle lengths (0.1, 1, 5, 10, 15 mm) as termination criteria. **(F)** Helix angle distribution in a basal, midcavity, and apical slab of the infarcted animal with zoomed tractography. **(G)** Ratio of positive to negative voxels in basal, midcavity, and apical segments. Remaining ISO-treated animals (*n* = 11) served as reference.

Myocardial microstructure was studied at a spatial resolution of 100 × 100 × 100 μm^3^ using DT-MRI at 7 T. By using an *ex vivo* approach without constraining influences such as motion, strain, and electrocardiogram (ECG) triggering, we aimed for full coverage of the entire LV with the highest possible image resolution and quality. On average, the case showed highly reduced mean diffusivity in the LV when compared to the remaining animals (Figures 4A,B). Mean diffusivity was found to be higher in apical (=infarcted) than in basal (=remote) segments (Figure 4C). Three-dimensional tractography showed that orientation coherence of myocardial fibers was maintained on a submillimeter scale, while increasing minimal tract lengths revealed increasingly sparse tract reconstruction (Figures 4D,E). Compared to the remaining ISO-treated animals, there was a reduced number of voxels with positive helix angle in the midventricular and apical segments resulting in a lower positive-to-negative helix angle ratio, whereas the proportion of fiber tracts with positive helix angle was higher in basal segments (Figures 4F,G).

## Discussion

Evaluation of cardiac function by noninvasive imaging tools is a cornerstone in the diagnosis and follow-up evaluation of both MI and heart failure. Yet, the underlying structural basis for cardiac function abnormalities often remains elusive. DT-MRI is an emerging imaging technique that facilitates three-dimensional reconstruction of the cardiac myofiber arrangement on a submillimeter scale [as comprehensively reviewed by Mekkaoui et al. (8)]. In the present report, DT-MRI revealed a pronounced disarray and loss of contractile fiber tract as microstructural correlates of impaired cardiac function in an uncommon case of MI. In accordance with histopathologic analysis, the disturbance of myofiber organization occurred predominantly in exactly those myocardial segments that showed impaired contraction/deformation.

Given that ISO's cardiotoxic effects are believed to be mediated by aggravating the mismatch between myocardial oxygen demand (positive inotropic effect) and supply (reduced coronary flow via positive chronotropic effects and consecutive shortening of the diastolic interval), the used experimental model may be considered as a preclinical correlate of type 2 MI (9). Indeed, there is clinical evidence that β-adrenergic agonists can induce characteristics of MI, although these effects are transient when treated accordingly (10).

Apical ballooning is also a key feature of stress cardiomyopathy (also known as Takotsubo syndrome), a transient acute heart failure syndrome putatively caused by the release of catecholamines in response to sympathetic stimulation (11). Interestingly, application of ISO has been shown to induce several characteristics of stress cardiomyopathy in rats including transient apical akinesia and reversible left ventricular systolic dysfunction (12). In contrast to stress cardiomyopathy, however, the present case showed an irreversible damage pattern with severe replacement fibrosis in response to cardiomyocyte death (apical scarring), a pronounced loss of myofiber tracts, and sustained left ventricular systolic failure. Typically, ISO leads to circumscribed subendocardial fibrosis in the used mouse model. As the damage exceeded the subendocardium and resulted in transmural affection in the presented case, it appears likely that (1) there was a higher vulnerability against ISO-mediated effects (e.g., greater response of the myocardium to β-adrenergic signaling); (2) ISO provoked unexpected thromboembolic coronary occlusion; and/or (3) ISO was accompanied by additional harmful effects, such as coronary artery dissection. However, our study was designed to characterize morphology and function rather than to elucidate the etiology of this unexpected event, which is why the prespecified study protocol did not include an appropriate assessment of the abovementioned aspects (e.g., lack of ECG recording and troponin assessment after ISO application, specialized tissue preparation for DT-MRI hampering the detection of thrombotic material).

In conclusion, this preclinical case report provides insights into pathophysiological and morphologic–functional relations of heart failure following MI by combining latest functional analysis and cardiac imaging techniques. The advent of clinical DT-MRI may facilitate simultaneous assessment of morphologic and functional changes under these conditions.

## Data Availability Statement

All datasets generated for this study are included in the article/Supplementary Material.

## Ethics Statement

The animal study was reviewed and approved by Landesamt für Gesundheit und Soziales (LAGeSo), Berlin, Germany.

## Author Contributions

NB performed data collection and statistical analyses, created the figures, and drafted the manuscript. DL performed data collection/data analysis related to DT-MRI and created individual figure tiles. AT, AF-L, and RK performed data collection. AF-L, LS, and UK conceived and designed the study, critically reviewed, and amended the manuscript. LS and UK acquired funding for the study. All authors have contributed extensively to the manuscript.

## Conflict of Interest

The authors declare that the research was conducted in the absence of any commercial or financial relationships that could be construed as a potential conflict of interest.

## Supplementary Material

Supplementary Video 1 Parasternal long-axis view at baseline prior to treatment. B-mode echocardiography showed normal left ventricular ejection fraction in the absence of any regional wall motion abnormalities.

Supplementary Video 2 Parasternal long-axis view 2 weeks after ISO treatment. B-mode echocardiography revealed reduced global systolic function due to a pronounced apical aneurysm. ECG-gated kilohertz visualization ultrasound imaging for ultrahigh spatiotemporal resolution.
